# Reproducibility of the CROSS Trial Results in the Multimodal Treatment of Esophageal Cancer in Daily Practice: A Single Center Retrospective Observational Study

**DOI:** 10.1155/2023/8955720

**Published:** 2023-01-30

**Authors:** H. Appius, J. Hafer, W. Harms, M. Bolli, M. Buess

**Affiliations:** ^1^Basel University, Basel, Switzerland; ^2^Department of Medical Oncology, University Hospital Basel, Basel, Switzerland; ^3^Radiation Oncology, St. Clara Hospital, Basel, Switzerland; ^4^Clarunis, Department of Visceral Surgery, University Center for Gastrointestinal and Liver Diseases, St. Clara Hospital and University Hospital Basel, Basel, Switzerland; ^5^Medical Oncology St. Clara Hospital and Basel University, Basel, Switzerland

## Abstract

**Background:**

Treatment of nonmetastatic esophageal cancer with curative intention remains a major challenge. Neoadjuvant radiochemotherapy followed by surgery, as described in the CROSS trial in 2012, has been established as a standard of care. With this retrospective observational study, we aimed to analyze the results of the CROSS regimen in daily practice over the last 10 years at the St. Clara Hospital, a Swiss center for esophageal surgery.

**Methods:**

To determine the clinical outcome in our daily practice, the medical records of all patients with potentially curable localized esophageal cancer (T1N1 or T2-3N0-1 M0) treated with radiochemotherapy in neoadjuvant intention according to the CROSS regimen were reviewed. The primary endpoint was overall survival. Furthermore, an overall survival analysis of the subgroups of patients who exactly met the inclusion criteria of the CROSS trial with respect to age and weight loss before therapy was performed. The Kaplan–Meier method was used to estimate survival and compared by the log-rank test.

**Results:**

From January 2012 to January 2022, 91 patients with T1N1 or T2-3N0-1M0 esophageal cancer underwent neoadjuvant radiochemotherapy according to the CROSS regimen. The median age was 70 years (range 31–86 years), and 26 (29%) patients were over 75 years of age. Weight loss of more than 10% was observed in 23 (25%) patients. 77 (85%) patients underwent esophagectomy, and complete resection (R0) was obtained in 73 (95%) of them. The median overall survival was 41 months, compared to 49.4 months in the CROSS trial. The overall survival rate at 12 months was 85% and at 24 months, it was 68%, very similar to the CROSS trial results. 51% of the patients fully met the inclusion criteria of the CROSS trial with respect to age and pretherapeutic weight loss. Their overall survival rate at 12 months was 94% at St. Clara Hospital versus 82% in the CROSS trial (*p*=0.04), and at 24 months, 81% versus 67% (*p*=0.05).

**Conclusion:**

Overall, in a center specialized for the multimodal treatment of nonmetastatic esophageal cancer, the results of the CROSS trial seem to be well reproducible in daily practice.

## 1. Introduction

Esophageal cancer is a major cause of cancer mortality across the world [1]. As in many developed countries, in Switzerland, a significant increase in adenocarcinomas of the esophagus was observed from 1986 to 2015 [2]. The simultaneous increase of gastroesophageal reflux disease and obesity might be at least partially responsible for this trend. The prognosis of metastatic esophageal cancer is dismal, and for nonmetastatic disease, the overall survival is unsatisfactory [3]. The treatment of nonmetastatic, potentially curable esophageal cancer still remains a major interdisciplinary challenge.

Since the publication of the CROSS trial in 2012, neoadjuvant radiochemotherapy (NARCT) with radiation to 41.4 Gy over 4.5 weeks in parallel with carboplatinum area under the curve (AUC) of 2 mg/ml/minute and paclitaxel 50 mg/m^2^ on days 1, 8, 15, 22, and 29 (CROSS regimen) has become the standard of care in nonmetastatic, resectable esophageal cancer [4]. The results of the CROSS trial were consistent over time and were confirmed after 5 and 10 years. The overall survival rates at 1, 2, 3, 5, and 10 years were 82%, 67%, 58%, 47%, and 38% versus 70%, 50%, 44%, 34%, and 25% [5, 6]. Clinical outcomes in daily practice, however, might differ significantly from the results of a prospective randomized trial. As expected, in Leyden University Medical Center, where the CROSS trial had been performed, the outcome in daily practice was slightly poorer than in the trial, mainly due to the presence of less favorable patient and tumor characteristics [7]. Furthermore, Wong et al. reported a much less favorable outcome in Asian patients, even for patients fulfilling the inclusion criteria of the CROSS trial [8].

With this retrospective observational study, we aimed to analyze the results of the CROSS regimen in daily practice over the last 10 years at the St. Clara Hospital, Basel, one of 8 centers in which esophageal cancer surgery is performed in Switzerland. Specific aims were to determine overall survival in daily practice for patients treated with the CROSS regimen who would have fulfilled the strict inclusion criteria of the CROSS trial and for patients who would have been excluded from the CROSS trial due to age >75 years or >10% loss of total body weight.

## 2. Methods

### 2.1. Participants and Data Collection

In this retrospective single-center observational study, the hospital administrative database was screened for patients treated with radiochemotherapy for esophageal cancer. Eligible participants were patients with tumors of clinical stage T1N1 or T2-3 N0-1 and with no evidence of metastatic spread (M0). Only data from patients with histologically confirmed adenocarcinoma or squamous cell carcinoma of the esophagus or esophagogastric junction were further analyzed. Other histologic subtypes were excluded. We included all patients between January 1, 2012, and January 31, 2022, who started neoadjuvant radiochemotherapy for esophageal cancer according to the CROSS regimen at St. Clara Hospital, Basel, Switzerland.

### 2.2. Baseline Evaluation

Baseline evaluation included upper gastrointestinal endoscopy, radiological staging with contrast-enhanced computed tomography of the thorax and abdomen or positron emission tomography-computed tomography (PET-CT). Endoscopy and biopsy were performed to determine the extent of tumor infiltration and for histological confirmation of the diagnosis. TNM-staging was performed using American Joint Committee on Cancer 7^th^ Edition [9]. Echocardiography and a pulmonary function test were performed to evaluate functional operability. Following initial clinical staging, all patients were discussed at a multidisciplinary tumor board, with treatment recommendations for NARCT followed by esophagectomy. All patients provided written informed consent before starting treatment.

### 2.3. CROSS Regimen

The NARCT schedule was as follows: on days 1, 8, 15, 22, and 29, carboplatin targeted at an AUC of 2 mg/ml/minute and paclitaxel at a dose of 50 mg/m^2^ of body surface area were administered intravenously. A total external beam radiotherapy (RT) dose of 41.4 Gray was given in 23 fractions (conventional fractionation) over 4.5 weeks, starting on the first day of the first cycle of chemotherapy. 3D conformal RT (3DCRT) or intensity-modulated RT (IMRT) was used to achieve adequate target coverage and adhere to the dose constraints for the organs at risk. During NARCT, patients were closely monitored for toxic effects. Toxicities were recorded using Common Terminology Criteria for Adverse Events (CTCAE) version 4 [10]. 3 weeks later, NARCT restaging with PET-CT was performed and the indication for operation was reevaluated at the interdisciplinary tumor board. 5 weeks after the end of NARCT, either an open or hybrid robot-assisted Ivor Lewis esophagectomy was performed.

### 2.4. Follow-Up

During radiochemotherapy, patients were seen every week in the outpatient clinic. After surgery, follow-up visits were scheduled every 3 months for the first year, then every 6 months up to 5 years. Imaging with a CT scan of the thorax and abdomen was recommended after 6, 12, 24, 36, 48, and 60 months. Follow-up after surgery was either performed in the oncology outpatient clinic or by a general practitioner.

### 2.5. Collection of Data

The following data were collected from electronic patient records: sex, age at diagnosis, date of death or date the patient was last known alive, date of diagnosis, tumor stage, tumor histology and location, relevant secondary diseases, weight at diagnosis, amount of weight loss before diagnosis, and the tumor board recommendation regarding therapy. If the initial recommendation was a NARCT with the CROSS regimen, other parameters were additionally collected, such as serious adverse events (SAE) > grade 2 during NARCT, functional operability, weight before surgery, date of surgery and procedure, if surgery was refused by the patient, postoperative tumor stage, tumor regression grading according to Mandard or Becker, R0 resectability, and whether adjuvant therapy was performed. Finally, it was determined whether there would be follow-up therapy. The study was approved by the local ethics committee on 11th October 2021 (EKNZ 21-01348).

### 2.6. Endpoints

The primary endpoint of this study was the overall survival of patients treated at St. Clara Hospital with neoadjuvant combined radiochemotherapy followed by surgery according to the CROSS regimen. Preplanned subgroup analysis were performed for the following patients: the overall survival in patients over 75 years compared to patients under 75 years, patients with weight loss over 10% or under 10%, and patients over 75 years or with more than 10% weight loss before diagnosis.

### 2.7. Statistical Analysis

All collected data were tabulated using Microsoft Excel. Survival was estimated from the start of treatment to the date of death, with censoring at the date of last follow-up contact for patients still alive. An intention-to-treat analysis was performed, including all patients who started neoadjuvant treatment with the CROSS regimen. The overall survival was analyzed using the Kaplan–Meier method with a two-sidedlog-rank test in GraphPad®. Among subgroups, discrete variables were compared using Fischer's exact in GraphPad®. To compare the survival rate at 1 and 2 years of the patients treated at the St. Clara Hospital with the survival rates in the CROSS trial, we formulated the following null hypothesis: the survival rate at 1 and 2 years at the St. Clara Hospital equals the survival rates in the CROSS trial. We performed a two-tailed*t*-test with the calculated survival rate at 1 and 2 years of the patients treated at the St. Clara Hospital, the standard deviation, the number of patients at risk at 1 and 2 years, and the reported survival rate at 1 and 2 years in the CROSS trial. *P* values of an alpha level of <0.05 were considered statistically significant.

## 3. Results

### 3.1. Study Population and Baseline Characteristics

**Figure 1:**
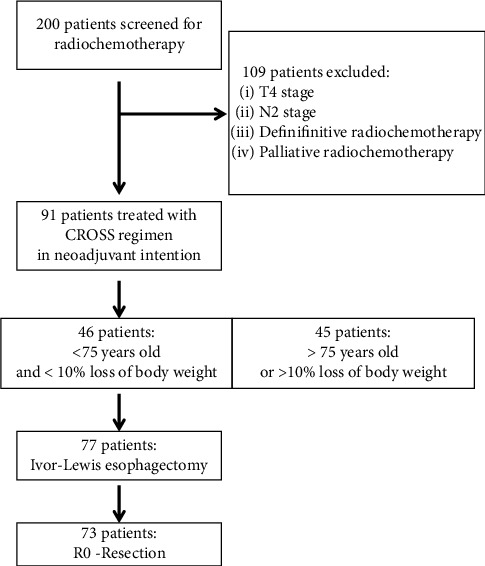
Flow diagram of patients included in the study by screening 200 electronic health records of patients diagnosed with esophageal cancer between 2012 and 2021 at the St. Clara Hospital and treated with neoadjuvant radiochemotherapy.

By screening the hospital administrative database for cases of esophageal cancer treated with combined radiochemotherapy between January 1, 2012, and January 31, 2022, 200 patients were identified. Of these patients, 109 were excluded due to tumor stage (cT4, cN2, cM1), a different treatment approach, or a different treatment goal (e.g., definitive radiochemotherapy or palliative radiochemotherapy). The remaining 91 patients with a cT1N1M0 or cT2-3N0-1M0 stage who started NARCT with the CROSS regimen formed the study population (Figure 1). Baseline characteristics are shown in Table 1. Patients had a median age of 75 years (33–86 years). 29% of them were over 75 years old. 77% were male patients. Most patients presented with a cT3 tumor stage (82%) and a cN1 nodal stage (65%). Adenocarcinomas dominated with 88% over squamous cell carcinomas with 12%. Gastroesophageal junction cancer was more frequent (87%) than intrathoracic cancer (13%). Loss of more than 10% of the total body weight was observed in 23 (25%) of the patients. Most patients (75%) had no relevant previous disease. 15% suffered from cardiac comorbidity.

### 3.2. Treatment

All patients underwent and completed radiotherapy as intended. 77% of the patients completed the full treatment regimen of 5 cycles of chemotherapy. 10% received 4 cycles, 6% received 3 cycles, and 6% received less than 3 cycles of chemotherapy. One patient did not receive any chemotherapy because of acute kidney failure. Reasons for discontinuation of chemotherapy were bizytopenia, esophagitis, infection, or an allergic reaction. The most common reason was thrombocytopenia grades 1 and 2 (13%).

Overall, 81 patients (89%) were functionally operable. Of these, four patients refused surgery, resulting in a total of 77 patients (85%) undergoing esophagectomy. Robot-assisted surgery was more common (53%) than open esophagectomy (47%). An R0 resection was achieved in 73 of 77 patients (95%). A postoperative tumor stage of ypT0 was reached in 18% of the patients. A complete nodal regression ypN0 was observed in 58%. The majority of the operated patients (89%) were evaluated according to Mandard's tumor regression system. The remainders were assessed using the tumor regression system of Becker. Increased residual cancer cells with predominant fibrosis (Mandard grade 3) were the most frequent pathological findings after esophagectomy with 30%, followed by grade 4 with 27%. 17% of patients who underwent esophagectomy received adjuvant therapy. No postoperative deaths occurred in the first 30 days. Follow-up therapy after relapse was given in 40% of patients, with palliative systemic therapy being the most common with 18%.

### 3.3. Toxicity of Radiochemotherapy

During neoadjuvant radiochemotherapy, the following adverse events ≥grade 2 were observed in 33% of patients: hemotoxicity (neutropenia 8% and thrombocytopenia 7%), radiation-induced esophagitis requiring hospitalization (7%), infections (especially pneumonia and catheter infection), and two patients with an allergic reaction.

### 3.4. Overall Survival

**Figure 2:**
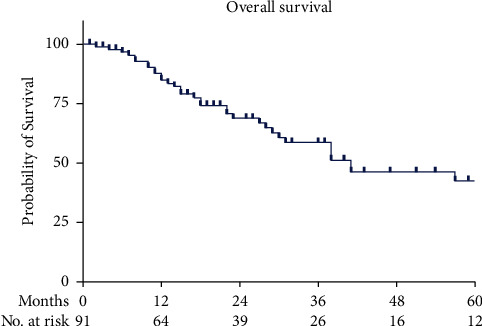
Kaplan–Meier plot of the estimated overall survival among patients with esophageal cancer who underwent neoadjuvant radiochemotherapy followed by surgery (*n* = 91). Tick marks indicate censored data.

To assess the results of neoadjuvant radiochemotherapy followed by surgery according to the CROSS protocol at the St. Clara Hospital and to compare the results with the CROSS trial, overall survival was used as the primary endpoint. The median follow-up was 32 months. The median overall survival (OS) time was 41 months in St. Clara Hospital compared to 49.4 months in the CROSS trial [4]. The OS rate over the first 2 years was similar between the patients of St. Clara Hospital and the patients of the CROSS trial. At 12 months, OS was 85% (95% CI: 77%–92%) compared to 82% in the CROSS trial, and at 24 months, 69% (95% CI: 61%–76%) versus 67%, respectively (Figure 2).

### 3.5. Overall Survival of Patients >75 Years of Age or with Major Weight Loss of >10% Body Weight

45 of the 91 patients (49%) who would have been excluded from the CROSS trial considering the age and amount of weight loss before diagnosis were nonetheless treated with the CROSS regimen. 26 patients were older than 75 years, and 19 patients presented with a weight loss of >10% of body weight. In four patients, both factors were found. Weight loss before diagnosis was more frequently observed in patients younger than 75 years (29% versus 15%).

**Figure 3:**
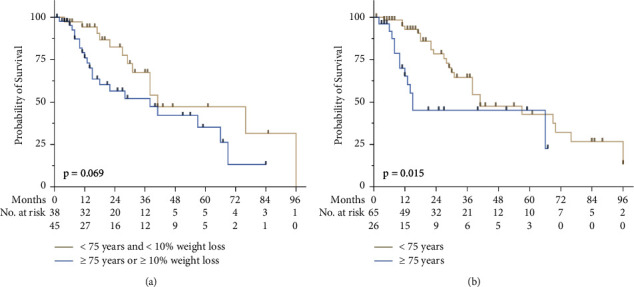
(a) Kaplan–Meier estimates of overall survival among patients with esophageal cancer at St. Clara Hospital who would have been excluded from the CROSS trial because they were ≥75 years or had ≥10% weight loss compared to patients who were in accordance with the CROSS trial inclusion criteria: <75 years and <10% weight loss. Tick marks indicate censored data. The group of patients who fully met the inclusion criteria showed a median overall survival of 41 months compared to 38 months in the group that did not meet the inclusion criteria of the CROSS trial, which was not statistically significant (HR: 1.9, *p*=0.069). (b) Kaplan–Meier estimates of overall survival among patient group ≥75 years or <75 years with esophageal cancer treated at St. Clara Hospital with neoadjuvant radiochemotherapy followed by surgery. Tick marks indicate censored data. Patients >75 years showed a significantly shorter median survival of 15 months compared to patients <75 years of age (41 months) (HR: 2.2, log-rank*p*=0.015).

Focusing on the patients who fully met the inclusion criteria of the CROSS trial by stage, age, and loss of weight, the OS rate at 12 months was 94% (95% CI: 83%–106%) at the St. Clara Hospital versus 82% in the CROSS trial (two-tailed*p*=0.04), and at 24 months, 82% (95% CI: 71%–92%) versus 67% (two-tailed*p*=0.05). The group of patients that fully met the inclusion criteria showed a median OS of 41 months compared to 38 months in the group that would not have met the inclusion criteria of the CROSS trial. This difference in OS was not statistically significant (HR: 1.9, log-rank*p*=0.069) (Figure 3(a)).

Patients >75 years of age had a significantly shorter median OS of 15 months than patients <75 years of age (41 months) (HR: 2.2, log-rank*p*=0.015) (Figure 3(b)). Patients >75 years of age presented with more cardiac comorbidities (7 out of 26 (27%)) than patients <75 years (7 out of 65 (11%)) (Fischer's exact *p*=0.1). Furthermore, there were more functionally inoperable patients in that age group (5 out of 26 (19%) vs. 4 out of 65 (6%), Fischer's Exact *p*=0.1). The differences, however, were not significant. Preoperative complications due to NARCT and chemotherapy dose reductions were similar in both groups. No difference in overall survival was seen between patients with major or minor weight loss. The median survival was 38 months in both groups (HR: 1.1, log-rank*p*=0.76).

## 4. Discussion

The trimodality approach with preoperative radiochemotherapy followed by surgery improved survival among patients with resectable, potentially curable esophageal cancer in the prospective randomized controlled CROSS trial [4]. Outside of the stringent selection of patients for a clinical trial, it remains unclear whether patients in clinical practice also derive the same benefit since patients with higher risks frequently undergo this treatment approach. We performed this study to determine the overall survival rate of patients from our daily practice. Our assumption was that the effects of neoadjuvant radiochemotherapy shown in the CROSS trial might be smaller in the “real-world scenario” due to the inclusion of patients bearing higher risks, such as a higher age and weight loss before therapy. In this single-center retrospective analysis, we found that median overall survival, the primary endpoint of the study, was numerically (41 versus 49.4 months) shorter than in the CROSS trial. Since the median follow-up time in our study was 32 months, the median overall survival of 41 months in our study has to be interpreted with caution. In the first two years, the overall survival rate was very similar to the randomized CROSS trial (85% versus 81% at 12 months and 69% versus 67% at 24 months), even though nearly half of the patients (49%) treated at St. Clara Hospital would have been excluded from the CROSS trial (Figure 1).

It is interesting to note that patients treated at the St. Clara Hospital who fully met the inclusion criteria of the CROSS trial had a significantly better overall survival rate at 12 months (94% versus 82%, *p*=0.04) compared to the CROSS trial patients with an even more pronounced difference at 24 months and an overall survival rate of 82% versus 67% (*p*=0.05) (Figure 2). The better outcome in the St. Clara Hospital cohort might partially be explained by lower postoperative mortality within the first 30 days (0% vs 2% in the CROSS trial). However, better results from a small, retrospective, nonrandomized study compared to a large, randomized trial raise the obvious question of whether there might be a bias. As in all retrospectively defined cohorts, substantial bias could be due to patients who were not included in the analysis. In our study, we screened our hospital database for radiochemotherapy in esophageal cancer. Therefore, we missed all patients who did not start radiochemotherapy. This differs from the CROSS trial, in which all patients were included and analyzed according to the intention-to-treat principle. 5% of them did not start radiochemotherapy [4]. These patients, for whom we can assume an unfavorable outcome, are missing from our cohort, which might partially explain the more favorable results in our cohort compared to the CROSS trial. A further difference from the CROSS trial might be the follow-up therapies after relapse, which can prolong the overall survival time. In the CROSS trial, they are not well defined. In our cohort, most patients received chemotherapy with palliative intention; some patients received radiochemotherapy or radiotherapy alone; and in the last months of the study period, a few patients obtained a newly available adjuvant immunotherapy. The effect of these therapies on the prolongation of overall survival cannot currently be well defined. Even under these circumstances, we conclude that the results of the CROSS trial are fully reproducible in our daily practice.

31% of patients with esophageal cancer are >75 years old. 67% of them have a potentially curable disease state, which is higher than in the younger age groups [11]. This is over 52% of the total potentially curable population. The CROSS trial excluded this large population. Even including these elderly patients (>75 years old) led to results, which were very similar to the results of the CROSS trial. However, focusing specifically on the elderly patients over 75 years, a significantly lower median overall survival of 15 months was observed compared to the study population of the CROSS trial, where such patients were excluded (Figure 3(b)). However, once these patients exceeded the first 12 months, their prognosis was very similar to that of the rest of the trial population. It appears that selected patients might benefit from the trimodal approach in the long term, while for other patients >75 years of age, this approach is not providing the expected benefit. Therefore, the question arises whether definitive radiochemotherapy instead of the trimodality approach might be preferable in patients older than 75 years or at least in a subgroup with additional comorbidities.

Patients with major and those with minor or no weight loss had an equal median overall survival of 38 months (log-rank *p*=0.76). It seems that the amount of weight loss is not relevant for overall survival in our cohort. This suggests that patients with weight loss >10% should not be excluded from the trimodality concept but should be well nourished preoperatively during NARCT to ensure optimal conditions for surgery.

A weakness and limitation of our retrospective study is the small sample size. Subgroup analyses have to be interpreted with the necessary caution, and several additional interesting questions remain open, e.g., the difference between the therapeutic results in adenocarcinomas versus squamous cell carcinomas. A further limitation is the short median follow-up time of less than three years. However, we think that we restricted the interpretation of the data to draw valid conclusions.

In summary, we have shown that the outcome of patients treated in daily practice at the St. Clara Hospital corresponds to the results of the CROSS trial. Patients who met all the inclusion criteria of the CROSS trial even had a significantly better outcome than those in the CROSS trial. In contrast, elderly patients over 75 years of age had a worse outcome, as reported in previous studies. The CROSS regimen shows similar efficacy in clinical practice at a specialized center for multimodality treatment of nonmetastatic esophageal cancer, and the results of the CROSS trial are fully reproducible in our daily practice.

## Figures and Tables

**Table 1:** Baseline characteristics of all patients with esophageal cancer diagnosed between 2012 and 2022 who underwent treatment with neoadjuvant radiochemotherapy at St. Clara Hospital (*N* = 91).

	*N* (%)
Age	
Median age year (range)	70 (33–86)
Age ≥75 years	26 (29)
Gender	
Male	70 (77)
Female	21 (23)
Clinical tumor stage	
cT1	1 (1)
cT2	15 (16)
cT3	75 (82)
Clinical nodal stage	
cN0	29 (32)
cN1	59 (65)
cNX	3 (3)
Clinical metastasis stage	
cM0	90 (99)
cMX	1 (1)
Tumor histology	
Adenocarcinoma	80 (88)
Squamous cell carcinoma	11 (12)
Tumor localization	
Gastroesophageal	79 (87)
Intrathoracic	12 (13)
Relevant comorbidity	
Cardiac disease	14 (15)
Kidney disease	2 (2)
No preexisting disease	68 (75)
Other malignancies	4 (4)
Pulmonary disease	2 (2)
Psychiatric disease/disease of the central nerve system	1 (1)
Weight loss	
≥10%	23 (25)
<10%	55 (60)
Could not be determined	13 (14)

## Data Availability

The datasets used and/or analyzed are available from the corresponding author upon request.
